# Study of Frequency and Characteristics of Red Blood Cell Alloimmunization in Thalassemic Patients: Multicenter Study from Palestine

**DOI:** 10.1155/2019/3295786

**Published:** 2019-11-12

**Authors:** Adham Abu Taha, Ahmad Yaseen, Sa'd Suleiman, Omar Abu Zenah, Hammam Ali, Rania Abu Seir, Khaled Younis

**Affiliations:** ^1^College of Medicine and Health Sciences, An-Najah National University, Nablus, State of Palestine; ^2^Department of Medical Lab Sciences, Faculty of Health Professions, Al-Quds University, Jerusalem, State of Palestine

## Abstract

*Background. β*-Thalassemia is a common inherited hemolytic disorder in Palestine. Red blood cell (RBC) transfusion is the principal treatment but it may cause RBC alloimmunization. This study was conducted to determine the prevalence and characteristics of RBC alloimmunization among thalassemic patients in northern governorates of Palestine. *Methods. *A prospective multicenter observational study was conducted in the thalassemia transfusion centers in the northern governorates of Palestine. The study included 215 thalassemia patients who received regular blood transfusions. Clinical and transfusion records of patients were examined. Antibody screening and identification was conducted using the microcolum gel technique. *Results. *Two hundred fifteen patients were included in the study. More than half (52.1%) of the patients were males. The median age of patients was 18 years (range: 12–24 years). The most frequent blood group was A (40.5%). Alloantibodies were detected in 12.6% of patients. Anti-D (33.3%), anti-K (25.9%) and anti-E (14.8%) were the most commonly isolated antibodies. There was no association between age, sex, starting age of transfusion, number of transfused units, history of splenectomy and alloimmunization. *Conclusions. *Anti-Rh and anti-K antibodies were common among this cohort of patients. Age, sex, starting age of transfusion, number of transfused units, and history of splenectomy could not predict the occurrence of alloimmunization.

## 1. Introduction

Beta thalassemia (*β*-thalassemia) is the most common inherited hemolytic disorder caused by partial or complete defect in globin chain [1, 2]. Patients with *β*-thalassemia-major need regular blood transfusions throughout life. Blood is usually administered every 2–5 weeks in order to maintain the hemoglobin level between 9.5 and 10.5 g/dl [3, 4]. 

Alloimmunization to red cell antigens is one of the important complications of chronic blood transfusion in addition to iron overload and transfusion-transmitted infections [5, 6]. It can complicate transfusion therapy by causing delayed transfusion reactions and difficulties in finding compatible blood which may lead to increased morbidity and mortality in transfusion-dependent patients [7–9]. 

Alloimmunization rates among multiply-transfused thalassemia patients range from 2.5% to 42% in different regions of the world. The most common RBCs alloantibodies reported were ones against the Rhesus (Rh) and Kell antigens [10, 11]. There is only one previously published report about alloimmunization in multiply-transfused patients in Palestine. Samarah et al. reported the frequency of alloimmunization among sickle cell disease patients (7.76%) and the most frequent antibody was anti-K followed by anti-E antibodies [12]. 

The objectives of this study were to [1] assess alloimmunization among thalassemia patients in the northern governorates of Palestine, [2] determine the specificity of detected antibodies, and [3] to assess the possible association between previously reported risk factors and the development of alloimmunization.

## 2. Methods

A prospective multicenter observational study was conducted in all thalassemia transfusion centers in the northern governorates of Palestine (West Bank). Palestine consists of northern and southern governorates (The West Bank and Gaza Strip, respectively).

## 3. Patients

The total number of transfusion–dependent thalassemic patients in the northern governorates is 500 (verbal communication from the TPFS–Palestine). In total, 215 thalassemic patients (*β*-thalassemia major and *β*-thalassemia intermedia) were conveniently selected and evaluated. Transfusion-dependent patients who received at least 10 blood transfusions, of any age or gender were included. Multiply transfused patients with known connective tissue disease or any other autoimmune diseases were excluded.

## 4. Data Collection

Demographic and clinical information such as age, gender, status of the spleen, age of starting transfusion and number of transfused units were obtained directly from the participants (or their legal guardians) or from their medical files.

## 5. Laboratory Methods

Blood samples were collected from all patients. Plasma was screened for the presence of alloantibodies by using commercial three-cell panel (ID-Diacell I-II-II, Bio-Rad, Switzerland). The antibody specificity of all positive samples was determined using a commercial 11-cell identification panel (ID-DiaPanel, Bio-Rad, Switzerland).

## 6. Statistical Analysis

Statistical analysis was performed using the Statistical Package for the Social Science (SPSS) software (version 23.0; SPSS Inc., Chicago, IL, USA). Analysis included descriptive statistics, frequency distribution, mean, and standard deviation calculations. The relationship between alloimmunization and age, starting age of transfusion, and number of transfusions was assessed by Mann–Whitney *U* test. Chi-Square or Fisher's exact test was used to assess the association between gender and splenectomy and risk of alloimmunization. All reported *p* values are two-sided, with a priori significance level of 0.05.

## 7. Ethical Considerations

Informed consent was obtained from patients or their legal guardians. The study was approved by Institutional Review Board committee at An-Najah National University, the research ethics committee at Al-Quds University and the Palestine Ministry of Health.

## 8. Results

### 8.1. Patient Characteristics

During the study period, 215 patients were interviewed. There were 112 (52.1%) male and 103 (47.9) female patients (Table 1). The mean age was 19.02 ± 10.2 with a range from 2 to 70 years (Table 2). One hundred ninety-two patients (89.3%) were Rh(D) positive. The most frequent blood type was A (87; 40.5%) followed by O (81; 37.7%), B (35; 16.3%), and AB (12; 5.6%). One hundred ninety-five patients (90.7%) had *β*-thalassemia major and twenty (9.3%) had *β*-thalassemia intermedia. Less than half (102; 47.4%) of the patients had their spleen removed (Table 1). The mean age of starting blood transfusion was 16.55 ± 27 years with a range of 1 month to 25 years. The mean number of received blood units was 158.8 ± 144.2 with a range of 10–676 units of packed red blood cells (Table 2).

### 8.2. Alloantibody Screening and Specificity

Twenty-seven patients (12.6%) were alloimmunized. Twenty-three patients had a single alloantibody (85.2%) and four patients (14.8%) had multiple antibodies each (three patients had two antibodies each and one had three antibodies). The most frequent antibody was anti-D (9; 33.3%) followed by anti-K (7; 25.9%), anti-E (4; 14.8%) and anti-Kpa, anti-C, anti-Jka (1; 3.7% each) (Table 3).

### 8.3. Assessment of Predictors for Alloimmunization

Age of patients, starting age of transfusion, number of transfused blood units, gender, and splenectomy were not significantly different among alloimmunized and nonalloimmunized patients (Table 4).

## 9. Discussion

This study was conducted to determine the frequency and specificity of alloantibodies among transfusion-dependent thalassemia patients in the northern governorates. On the other hand, we assessed the association of previously reported risk factors and the development of alloimmunization in our cohort.

This is the first study to assess the frequency of alloantibodies among thalassemia patients in Palestine. The rate of alloamunization was 12.6%. Higher frequency of alloimmunization was reported from the province of Alexandria in Egypt (42.5%) [11], Taiwan (37%) [13], Kuwaiti Arabs (30%) [14], Saudi Arabia (22.06%) [15], Egypt (22.8%, 19.5% in limited donor program) [16], India (18.8%) [17] and Iran (17.9%, 16.3%); [18,19].

Other studies reported lower frequency of alloimmunization among transfusion-dependent thalassemia patients. Albania (11.8%); [20], Oman (9.3); [21], Malaysia (8.6%); [8], Pakistan (8.6%); [22], Jammu region in India (8.5%); [23], Fayoum province in Egypt (7.98%); [24], Tunis (7.7%); [25], southern Iran (5.3%); [26], Iraqi Kurdistan (4.5%); [27] and Karachi-Pakistan (3.75%); [28].

The most frequent alloantibodies were anti-D, anti-K and anti-E (33.3%, 25.9%, and 14.8% respectively). This finding is similar to those reported in Egypt, India, and Pakistan, where antibodies against the Rh-system and Kell system antigens [22, 27, 29, 30] were the most frequent. In a study done by Ameen et al. [14], it was found that alloantibodies against antigens from the Kell-system are the most common alloantibodies followed by those against the Rh-system in alloimmunized transfusion-dependent Arab thalassemia patients in Kuwait, where anti-K was the most common followed by anti-E [14]. Moreover, Karimi et al. [26] and Davari in Iran [19] reported that alloantibodies belonging to the Kell-system were the most common alloantibodies. A lower anti-K frequency of 0.53% was reported in Fayoum, Egypt [24]. 

Predictors of alloimmunization among multiply transfused patients are still debatable. Our study showed no significant association with age and this is in agreement with Al-Mousawi et al. [27] and Elhence et al. [31]. On the other hand Al-Riyami et al. from Oman reported a significant association between age and alloimmunization [21]. 

Alloimmunization in splenectomized patients in our study was not statistically different from nonsplectomized patients. Several studies reported insignificant association between splenectomy and alloimmunization [21, 26, 27, 30, 31]. Other studies reported a significant association [24, 29, 32].

Age at initiation of transfusion was not significantly different among alloimmunized and non-alloimmunized patients. This is in agreement with Karimi et al., Amin et al., and Elhence et al. [18, 26, 31]. Other reports showed a significant association [24, 27].

Contrary to other reports [21, 23, 26, 29, 32], we did not find a significant association between the numbers of transfused blood units the development of alloimmunization and this is in agreement with Al-Mousawi et al. [27].

Similar to our study, few reports did not find a significant relationship between alloimmunization and gender [21, 23, 27]. Other reports found female gender [24, 33] or male [29] as a risk factor for alloimmunzion.

## 10. Limitations of the Study

The incidence of thalassemia in Palestine is dwindling and this is due to the premarital screening program enforced by the Palestine Ministry of Health for beta thalassemia carriers and discouraging marriage between carriers. Clinical data were collected from a computerized system in the Palestinian Ministry of Health, but some data such as age of starting transfusion, transfusion frequency and number of transfused units was unavailable. Therefore, age of starting transfusion and transfusion frequency were estimated by patients or guardians and the number of transfused units was estimated by calculations from the age of starting transfusion and transfusion frequency.

## 11. Conclusions

The high frequency of anti-D antibodies highlights the need to implement strict quality control programs in the local blood banks to test for weak D positive antigens. Red cell alloimmunization in transfusion dependent thalassemia patients can be minimized by phenotyping for Rh and Kell system.

## Figures and Tables

**Table 1:** Patients' characteristics.

Variable	Frequency	Percentage
*Gender*		
Male	112	52.1
Female	103	47.9
*Diagnosis*		
Beta thalassemia major	195	90.7
Beta thalassemia intermedia	20	9.3
*Blood group*		
A	87	40.5
B	35	16.3
O	81	37.7
AB	12	5.6
*Rh (D)*		
Positive	192	89.3
Negative	23	10.7
*Splenectomy*		
Yes	102	47.4
No	113	52.6
*Antibody screening*		
Positive	27	12.6
Negative	188	87.4

**Table 2:** Transfusion history.

	Mean ± SD	Range
Age	19.02 ± 10.2	2–70
Transfusion starting age (month)	16.55 ± 27	1–300
Number of transfusions	158.8 ± 144.2	10–676

**Table 3:** Antibody specificity.

Type	*n* (%)
Anti-D	9 (33.3)
Anti-K	7 (25.9)
Anti-E	4 (14.8)
Anti-C	1 (3.7)
Anti-Kpa	1 (3.7)
Anti-Jka	1 (3.7)
Anti-E & anti-K	1 (3.7)
Anti-E & anti-Jka	1 (3.7)
Anti-C & anti-D	1 (3.7)
Anti-E, anti-Jka & anti-K	1 (3.7)
Total	27 (100)

**Table 4:** Contribution of potential of risk factors to alloimmunization.

Variable	Alloimmunized median (Q1–Q3) or *n* (%)	Nonalloimmunized median (Q1–Q3) or *n* (%)	*p*-value
*Age (years)*	18 (12–24)	18 (12–24)	0.832
Transfusion starting age (month)	12 (6–12)	12 (5.3–15.5)	0.875
Number of transfusions	107 (71–329)	102 (68–200)	0.42
*Gender*			
Male	10 (37.0)	102 (54.3)	0.103
Female	17 (63.0)	86 (45.7)	
*History of splenectomy*			
Yes	11 (40.7)	91 (48.4)	0.539
No	16 (59.3)	97 (51.6)	

## Data Availability

Data supporting the findings will be provided upon request.
